# Electrospun Poly(L-Lactic Acid)/Gelatin Hybrid Polymer as a Barrier to Periodontal Tissue Regeneration

**DOI:** 10.3390/polym15183844

**Published:** 2023-09-21

**Authors:** Youngchae Cho, Heeseok Jeong, Baeyeon Kim, Juwoong Jang, Yo-Seung Song, Deuk Yong Lee

**Affiliations:** 1 Department of Biomedical Engineering, Daelim University, Anyang 13916, Republic of Korea; youngchae6744@naver.com (Y.C.); hsjeong@daelim.ac.kr (H.J.); 2 Department of Materials Science and Engineering, Incheon National University, Incheon 22012, Republic of Korea; bykim@incheon.ac.kr; 3 Department of R&D Center, Renewmedical Co., Ltd., Bucheon 14532, Republic of Korea; orienta@empas.com; 4 Department of Materials Science and Engineering, Korea Aviation University, Goyang 10540, Republic of Korea; yssong@kau.ac.kr

**Keywords:** absorbable periodontal tissue regeneration, poly(L-lactic acid), gelatin, electrospinning, barrier membrane

## Abstract

Poly(L-lactic acid) (PLLA) and PLLA/gelatin polymers were prepared via electrospinning to evaluate the effect of PLLA and gelatin content on the mechanical properties, water uptake capacity (WUC), water contact angle (WCA), degradation rate, cytotoxicity and cell proliferation of membranes. As the PLLA concentration increased from 1 wt% to 3 wt%, the tensile strength increased from 5.8 MPa to 9.1 MPa but decreased to 7.0 MPa with 4 wt% PLLA doping. The WUC decreased rapidly from 594% to 236% as the PLLA content increased from 1 to 4 wt% due to the increased hydrophobicity of PLLA. As the gelatin content was increased to 3 wt% PLLA, the strength, WUC and WCA of the PLLA/gelatin membrane changed from 9.1 ± 0.9 MPa to 13.3 ± 2.3 MPa, from 329% to 1248% and from 127 ± 1.2° to 0°, respectively, with increasing gelatin content from 0 to 40 wt%. However, the failure strain decreased from 3.0 to 0.5. The biodegradability of the PLLA/gelatin blend increased from 3 to 38% as the gelatin content increased to 40 wt%. The viability of L-929 and MG-63 cells in the PLLA/gelatin blend was over 95%, and the excellent cell proliferation and mechanical properties suggested that the tunable PLLA/gelatin barrier membrane was well suited for absorbable periodontal tissue regeneration.

## 1. Introduction

Poly(lactic acid) (PLA), poly(L-lactic acid) (PLLA), poly(D-lactic acid) (PDLA), poly(D,L-lactic acid) (PDLLA), poly(lactic-*co*-glycolic acid) (PLGA) and poly(ε-caprolactone) (PCL) can be applied as biodegradable scaffold materials for environmental and tissue engineering because of their mechanical properties and biodegradability [1,2,3,4,5,6,7,8,9,10,11,12,13]. Although biodegradable polymers (BPs) have many advantages, such as morphological similarity to the extracellular matrix (ECM) of the native tissues and good cell adhesion and proliferation, the hydrophobicity of BPs limits their widespread use [7,8,9,10,11,12,13,14,15,16,17,18,19,20,21,22]. The hydrophobicity of electrospun BP can be tuned with surface modification via sputtering [8], the hydrothermal route [9] using hydrophilic materials or by alloying with natural polymers at the macromolecular level [1,2,3,4,5,6,7,10,11,12,13]. Plasma treatment of the surface of a BP can improve wettability and biocompatibility for guided periodontal tissue regeneration (GTR). However, surface modification via sputtering occurs near the surface and hydrophobic fibers located deep in the material remain intact [8]. The wettability and mechanical properties of BPs were also improved using a hydrothermal process (PLA coated with poly(vinyl alcohol) (PVA)), but studies are needed on the in vivo biocompatibility and biodegradability [9]. BP/gelatin hybrid membranes with specific ratios of BP and gelatin also exhibit tunable hydrophilicity, mechanical properties and biological functions [2,3,4,6,7,17]. Non-toxic aqueous solvents have emerged as solvents for dissolving and electrospinning natural polymers [4,6,16,20,21,22,23]. The poor mechanical properties of hybrid polymers prepared using green solvents are most likely due to the slow reaction rate of aliphatic polymer chains [24]. Because fluorine-based alcohol (1,1,1,3,3,3-hexafluoro-2-propanol (HFIP)) has strong functionality, it can cause a quick reaction to improve the mechanical properties of hybrid membranes [4,7]. The fluorine in HFIP attracts the negative charge of the conjugate base through a sigma bond and delocalizes the electron density, making it a strong acid. Fast chemical reactions can improve properties [4,7].

Gingivitis refers to inflammation of the gums [1,5,7,18,19]. It is a relatively mild disease in which the inflammation is confined to the gums and does not spread to the periodontal ligament or periodontal bone. If the oral cavity is not clean, bacteria in the plaque multiply and gingivitis occurs. When gingivitis gets worse, the inflammation spreads to the area around the gum bone, which is called periodontitis [18,19]. It is a chronic disease that is caused by bacteria and toxins in the plaque that inflame the supporting tissue surrounding the teeth. The periodontal tissue that supports the teeth is slowly destroyed, causing the teeth to loosen and eventually fall out. Periodontal regeneration is known as the regeneration of tissues supporting teeth, such as cementum, periodontal ligament and alveolar bone [1,18,19]. A bone graft material is used to regenerate the alveolar bone and the attachment between the tooth and the alveolar bone, and an absorbable periodontal barrier film is used in the form of a shield to protect the bone graft material from the invasion of unwanted tissue intrusion [1,11,18]. An absorbable barrier membrane is widely used in dentistry because it does not require secondary surgery compared with a non-absorbable Ti membrane [9,20,25,26]. Synthetic implant materials used to induce GTR use biocompatible polymers and are mainly manufactured using 3D printing or electrospinning [1,2,4,7]. In this study, the hydrophilicity and cell proliferation of BP were enhanced via alloying with natural polymers [1,4,5,6,7]. PLLA/gelatin membranes composed of biopolymers and natural polymers play an important role in tissue engineering due to their tunable mechanical properties, biocompatibility and biodegradability [6,7]. Nanofibrous PLLA/gelatin membranes with specific ratios of PLLA and gelatin were examined using HFIP as a solvent. The effects of polymer concentration on the mechanical properties, WUC, degradation rate and cytotoxicity of PLLA/gelatin membranes were investigated to demonstrate the feasibility of PLLA/gelatin membranes as a barrier for absorbable periodontal tissue regeneration.

## 2. Materials and Methods

### 2.1. Materials

PLLA (Resomer^®^ L210S, inherent viscosity 3.3~4.3 dL/g, St. Louis, MO, USA) and gelatin from porcine skin (gel strength 300, Type A, St. Louis, MO, USA) were purchased from Sigma-Aldrich, USA. HFIP (Daejung Chemicals & Metals Co., Ltd., Cheongju, Chungbuk, Republic of Korea) was used as the solvent for PLLA/gelatin throughout the study. All chemicals were used as received.

### 2.2. Electrospinning

**Figure 1:**
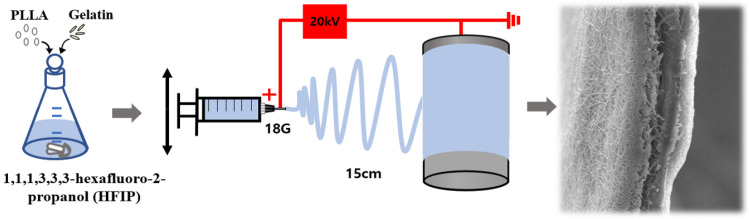
Illustration of the experimental setup.

The electrospinning apparatus consisted of a syringe pump (KDS-200, Stoelting Co., Wood Dale, IL, USA), a BD metal needle, a grounded collector, and a high-voltage power supply (ES30P-5W, Gamma High Voltage Research Inc., Ormond Beach, FL, USA) equipped with current and voltage digital meters [7,23,27]. The PLLA/gelatin precursor solution was placed in a 20 mL BD luer-lock syringe attached to a syringe pump (EP100, NanoNC Co., Ltd., Seoul, Republic of Korea) and fed to an 18-gauge metal needle at a flow rate of 1 mL/h. PDLLA/gelatin membranes were collected at a voltage of 20 kV and a distance of 15 cm using a rotating metal drum with a diameter of 9 cm and a length of 20 cm, as shown in Figure 1. The rotational speed of the mandrel-type collector and the transverse speed of the needle were 250 rpm and 60 cm/min, respectively [7]. PLLA in the range of 1 to 4 wt% was prepared by dissolving in HFIP using a magnetic stirrer for 6 h at room temperature. Gelatin (0~40 wt%) was then added to the PLLA solution. The precursor solution viscosity was examined at room temperature using a viscometer (DV 1M, Brookfield, Middleboro, MA, USA). The electrospun membrane was dried for 24 h in air, rinsed in distilled water for 5 min, dried for 24 h and finally vacuum dried for 48 h at 37 °C to remove any residual solvent.

Fourier-transform infrared spectroscopy (FT-IR, Spectrum Two, PerkinElmer, Beaconsfield, UK) was performed to investigate the chemical bonding of the membrane [25,26,27,28,29]. The morphology of the membrane was examined by using an SEM (S-3000H, Hitachi High-Tech Co., Tokyo, Japan) and an optical stereomicroscope (SV-55, Sometech Inc., Seoul, Republic of Korea). Prior to the SEM measurement, the surface was coated with Au-Pd for 120 s using an ion sputter (E-1010, Hitachi Science Systems, Ltd., Tokyo, Japan). The fiber diameter was measured using an optical microscope equipped with *IMT iSolution Lite image* software (ver. 9.1, IMT i-Solution Inc., Vancouver, BC, Canada) [27]. The tensile strength of the membrane was tested using a universal testing machine (Instron 5564, Norwood, MA, USA) with a crosshead speed of 10 mm/min. Specimens were prepared in dumbbell shape according to ASTM D-638 (type V). All experiments were performed at least 5 times. The WUC of the membrane was determined using the equation of $WUC\left(\%\right)=\frac{W_{f}-W_{i}}{W_{i}}\times 100$, where *W_i_* and *W_f_* are the weights of the dry sample and sample after immersion in distilled water for 24 h at room temperature, respectively [28,29]. The porosity of the membrane was examined using the liquid displacement technique [28]. The lyophilized samples were cut into 10 × 10 mm^2^ pieces and weighed before being immersed in 0.01 M PBS containing 0.1 mg/mL of lysozyme (Muramidase from hen egg white, Roche Diagnostics GmbH, Mannheim, Germany) at 37 °C. The samples were incubated to simulate the body environment. The specimens were taken out every 7 days and thoroughly freeze-dried. The degradation rate (DR) was determined using the equation of $DR\left(\%\right)=\frac{W_{i}-W_{f}}{W_{i}}\times 100$, where *W_i_* and *W_f_* are the initial weight of the sample and the final weight after removal from the medium, respectively [28]. The water contact angle (WCA) was measured to examine the hydrophilicity of the fabricated electrospun membrane. The contact angle analysis was performed using a droplet analysis device (SmarDrop Standard, FEMTOFAB, Seongnam, Gyeonggi-do, Republic of Korea). A total of 2 μL of distilled water was dropped in each sample. After 5 s, the static contact angle was measured and analyzed according to the instrument’s software [8]. The values in this text are expressed as the mean ± standard deviation, and *p* < 0.05 was considered statistically significant [23,27]. Tukey’s test using IBM SPSS software (version 23.0, IBM Co., Armonk, NY, USA) and one-way analysis of variance (ANOVA) were used to determine the significance of the differences [7].

### 2.3. Cytotoxicity and Cell Proliferation

The extract test method was conducted on the PLLA/gelatin membranes to evaluate the potential of cytotoxicity on the base of the International Organization for Standardization (ISO 10993-5) [27,30,31]. Membranes were extracted aseptically with serum in a single-strength Minimum Essential Medium (1× MEM, Dulbecco’s Modified Eagle’s Medium (Gibco) with 10% fetal bovine serum (Gibco) and 1% penicillin–streptomysin). The ratio of membranes to extraction vehicle was 6 cm^2^/mL (ISO 10993-12) [32]. After incubation for 24 h, the test extracts were placed on separate confluent monolayers of L-929 (NCTC Clone 929, ATCC, Manassas, VA, USA) and MG-63 (Korea Cell Line Bank, Seoul, Republic of Korea). Detailed experimental procedures are described elsewhere [23,27,30].

Cell Counting Kit-8 (CCK-8, Dojindo Molecular Technologies, Inc., Tokyo, Japan) was used for the assay of cell proliferation. CCK-8, being nonradioactive, allows for sensitive colorimetric assays for the determination of the number of viable cells in cell proliferation [23,27,30]. Water-soluble tetrazolium salts (WSTs) are reduced by dehydrogenases in cells to give an orange product (formazan), which is soluble in the tissue culture medium. The amount of the formazan dye generated by dehydrogenases in cells is directly proportional to the number of living cells. The 96-well plate containing 100 μL of cell suspension (5 × 10^3^ cells/well) was incubated for 24 h at a temperature of 37 °C in a 5% CO_2_ atmosphere. The test extracts (10 μL) were added to the plate and maintained for an appropriate length of time (6, 12, 24, 48 h) in a CO_2_ incubator. After adding 10 μL of CCK-8 solution to each well of the plate, the plate was incubated for 2 h. Then, the absorbance of the colored solution was quantified by measuring at a wavelength of 450 nm with the iMark microplate absorbance spectrophotometer (Bio-Rad, Hercules, CA, USA). The morphological change in the cell was examined to assess the biological reaction by using the inverted microscope (TS100-F, Nikon, Tokyo, Japan).

Each L-929 and MG-63 cell was seeded at 2 × 10^4^ cells in the 24-well plates. After that, the test extracts were added to the plate and maintained for an appropriate length of time (6, 12, 24, 48 h) in a CO_2_ incubator. The viability of live cells was determined using the Live/Dead Viability/Cytotoxicity Kit for mammalian cells (Thermo Fisher Scientific, Waltham, MA, USA). The images were observed using a fluorescence microscope (EVO FL, Thermo Fisher Scientific, Waltham, MA, USA) [33].

## 3. Results

### 3.1. PLLA Membrane

**Figure 2:**
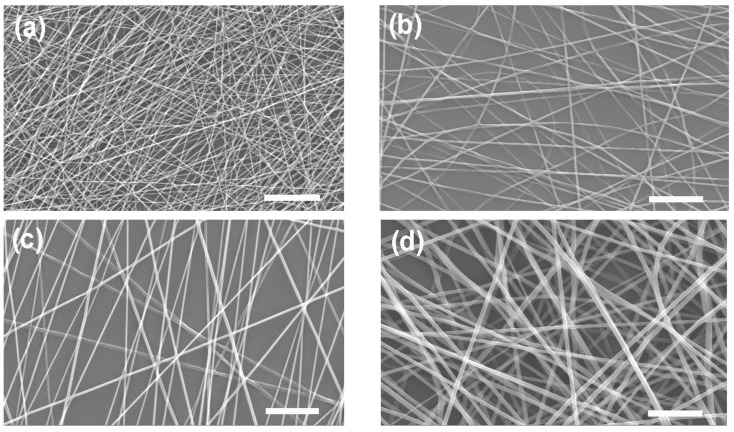
SEM images (2000×) of electrospun PLLA membranes as a function of PLLA concentration: (**a**) 1 wt%, (**b**) 2 wt%, (**c**) 3 wt% and (**d**) 4 wt%, respectively. Note that the scale bar is 10 μm.

The morphology of the PLLA membranes was examined using SEM, as shown in Figure 2. It shows a typical SEM morphology of a uniform, continuous PLLA fiber without beads. As the PLLA concentration increased from 1 wt% to 4 wt%, the viscosity increased from 45 cP to 1870 cP, resulting in a dramatic increase in the fiber diameter from 236 nm to 786 nm. The tensile strength and failure strain of the PLLA membranes as a function of PLLA concentration are shown in Figure S1. As the PLLA concentration increased from 1 wt% to 3 wt%, the tensile strength increased from 5.8 MPa to 9.1 MPa but decreased to 7.04 MPa with 4 wt% PLLA doping. The WUC decreased rapidly from 594% to 236% as the PLLA content increased from 1 wt% to 4 wt% due to the hydrophobicity of PLLA [2,4,7,26,34,35]. In this study, 3 wt% PLLA was selected as the optimal composition. The strength, strain at break and WUC of the 3 wt% PLLA membrane were measured to be 9.1 ± 0.9 MPa, 3.0 and 329%, respectively.

### 3.2. PLLA/Gelatin Membrane

**Figure 3:**
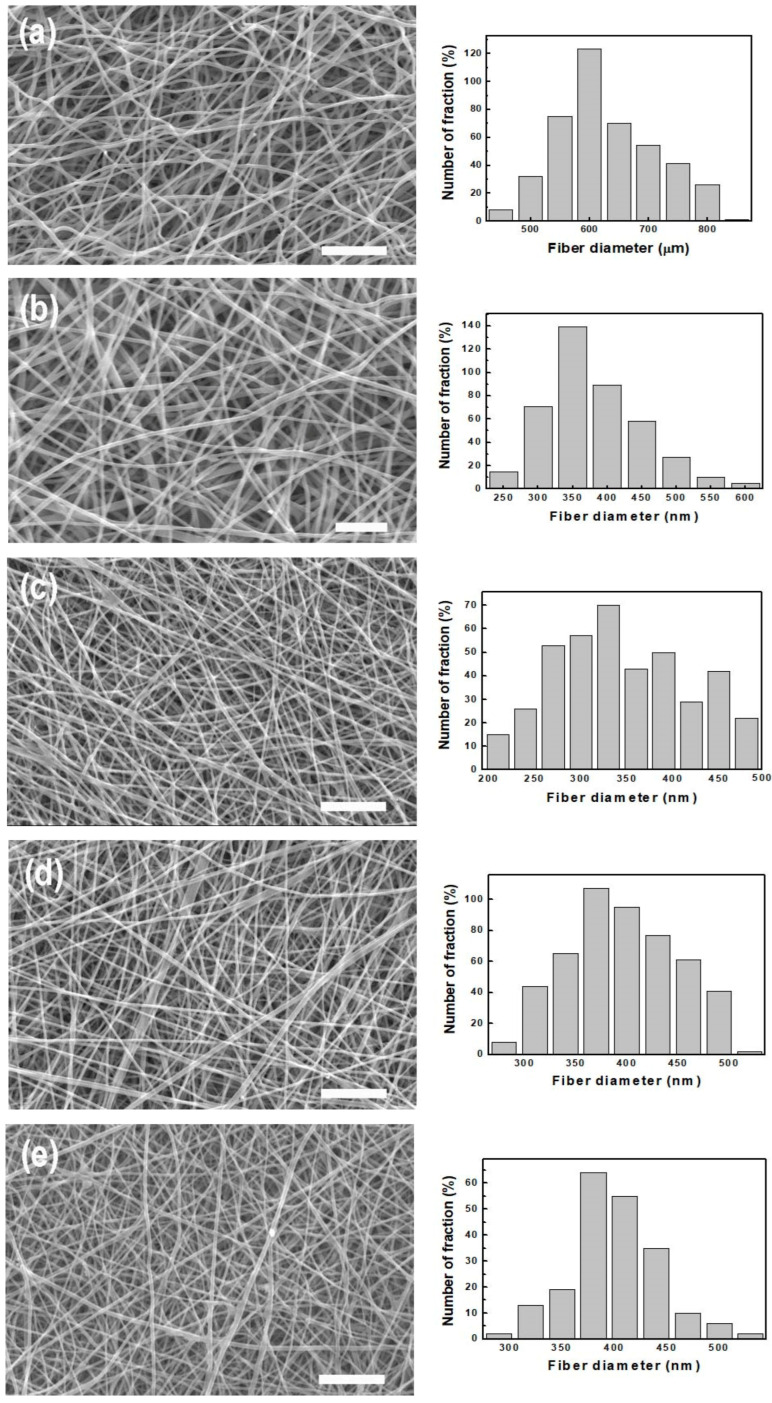
SEM images (2000×) of electrospun PLLA/gelatin membranes as a function of gelatin concentration: (**a**) 0 wt%, (**b**) 11 wt%, (**c**) 25 wt%, (**d**) 30 wt% and (**e**) 40 wt%. Note that the scale bar is 10 μm.

As a synthetic graft membrane, the absorbable commercial membrane (NeoDura) manufactured by Medprin Biotech GmbH (Frankfurt, Germany) is widely used. The strength, strain at break and WUC of NeoDura composed of PLLA and collagen were reported to be 3.7 ± 0.5 MPa, 0.55 and 455%, respectively [7]. The lower WUC of PLLA compared with NeoDura was addressed by alloying 3 wt% PLLA with gelatin (0 to 40 wt%). As the gelatin concentration increased from 0 wt% to 40 wt%, the tensile strength and WUC increased from 9.1 ± 0.9 MPa to 13.3 ± 2.3 MPa and from 329% to 1248%, respectively. However, the failure strain decreased dramatically from 3.0 to 0.5, as depicted in Figure S2. The modification of PLLA with other natural polymers can obviously increase the WUC with reduced failure strain [1,2,3,4,5,6,7,17,24]. Due to the contribution of gelatin in PLLA/gelatin membranes, a dramatic increase in the WUC was observed with increasing gelatin concentration [7]. Gelatin molecules with high dielectric constants are likely to become charged during electrospinning. Electrospinning jets with higher gelatin content are therefore more likely to have higher excess charges, resulting in thinner fibers [4,7,22]. Reducing the fiber diameter from 640 nm (PLLA) to 550 nm (PLLA/40 wt% gelatin) increased the porosity from 87% to 99%, as shown in Figure 3, increasing the WUC but reducing the failure strain. As the gelatin content increased from 0% to 11%, the strain at break decreased from 3.0 to 1.5, but WUC increased dramatically from 329% to 907%. With the addition of 40% gelatin, the WUC of the PLLA membrane increased from 329% to 1467%. However, for PLLA/gelatin membranes with gelatin doping above 25%, the strain to failure was dramatically reduced to less than 1.0. The reduced fiber diameter and the increased porosity may be responsible for the high swelling rate and poor ductility. Reduced deformation of the PLLA/gelatin barrier between the gingiva and alveolar bone may increase the risk of failure during suturing [1,5,6,7,26].

### 3.3. Wet Properties of PLLA/Gelatin Membrane

After surgery, the membrane must be anchored to the tissue to prevent bleeding and body fluid leakage and to achieve wound closure [36,37]. Sutures are traumatic to soft connective tissues, such as liver, spleen, kidney and lung. Laser tissue welding [35] and nanoparticle-based wound gluing [37] have been developed, enabling strong adhesion and permanent hemostasis within 1 min in situations with heavy blood flow. Both prevent new wound formation compared with conventional sutures. Because dental procedures in infants and young children are time-consuming and require little patience, the use of polymer adhesive is attractive. However, in adults, the absorbable periodontal barrier has typically been secured to the tissue using sutures, bone screws, or bone tacks because the surgical time and blood loss are not significant. Commercial periodontal barrier membranes and sutures were soaked in saline prior to use. In many cases, the firmness is lost in a wet state, and thus, there is a disadvantage in that space loss between the barrier membrane and the teeth may occur, which may adversely affect clinical results. Since the periodontal regeneration barrier membrane is used in a wet state, the wet state is more important than the dry state. If the membranes were not well wetted, the saline solution was allowed to absorb for 3 min by pressing with a gloved finger. The 3 wt% PLLA membrane soaked in water did not wet without finger pressure due to its hydrophobicity, whereas the gelatin-blended PLLA membranes were completely wetted, as displayed in Figure S3. After the water droplet was in contact with the 3 wt% PLLA membrane surface for 5 s, the WCA value for the PLLA was 127 ± 1.2°, which means that PLLA was highly hydrophobic. However, as the gelatin was blended with PLLA, the water droplets were completely absorbed, resulting in a WCA of 0° for the PLLA/gelatin membranes (Figure S3) [8]. Finger-stretched optical photographs of various PLLA/gelatin membranes in dry and wet conditions are shown in Figure S4. In the dry state, the extension properties of the PLLA/gelatin membrane decreased dramatically with increasing gelatin concentration (Figure S4), which is in good agreement with the previous strain results upon failure, as demonstrated in Figure S2. Since the PLLA membrane was hydrophobic, it was forcibly wetted with water using the force of a gloved finger. Figure S5 shows that there was no appreciable difference in the strain to failure between the dry and wet PLLA membranes. The strain of PLLA/gelatin membrane in the wet state increased from 0.5 to 2.1 as the gelatin was blended with PLLA at 40 wt%. Gelatin can hold large amounts of water within its network without dissolving or disrupting the structural integrity. Also, fragmented PLLA oligomers and monomers tend to crystallize inside the PLLA matrix because the polymer chains have sufficient mobility to rearrange into more stable configurations [14,15,38,39,40,41]. However, the tensile stress showed the opposite trend as the PLLA/gelatin membrane was wetted. During hydrolysis (Figure S6), water molecules are preferentially incorporated into the amorphous region, which enhances the polymer crystallinity because chain scission reactions are favored within amorphous regions [14,15]. The increase in failure strain of the PLLA/gelatin membrane could be attributed to the structural integrity of the gelatin and the improved PLLA crystallinity, but the initiation of ester bond cleavage due to hydrolysis is responsible for the reduction in stress [14,15,38,39,40,41]. However, the wet stress of the PLLA/gelatin membrane exhibits similar stress values regardless of the gelatin content because the soaking time was only 3 min and the degradation time was not sufficient.

### 3.4. Biodegradation

**Figure 4:**
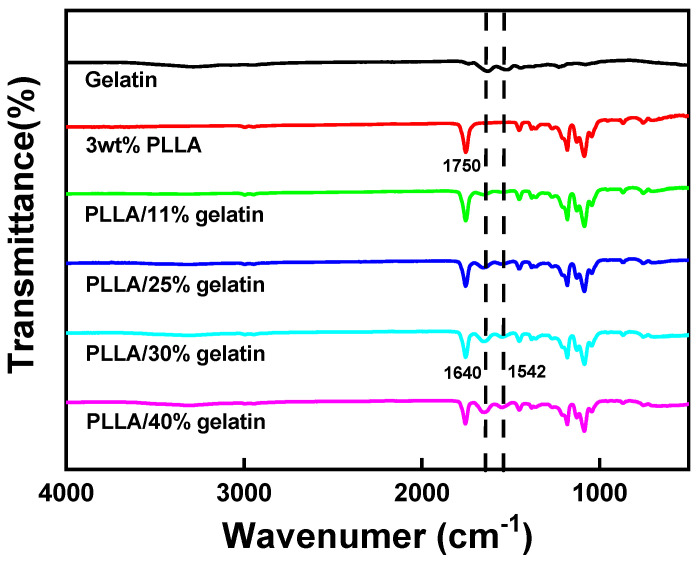
FT-IR spectra of gelatin, PLLA and PLLA/gelatin membranes.

**Figure 5:**
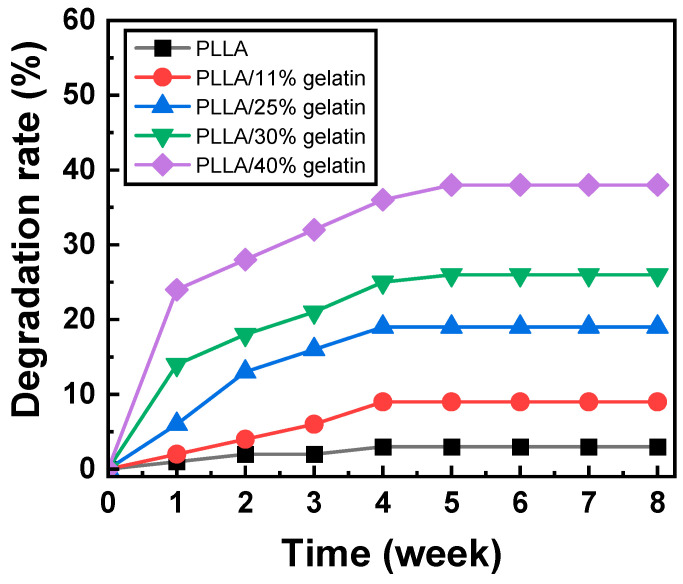
Enzymatic degradation rate of PLLA/gelatin membranes as a function of gelatin concentration.

FT-IR spectra of PLLA and PLLA/gelatin membranes are shown in Figure 4. As a PLLA structure, the peaks of the carbonyl group of aliphatic esters (-C=O, 1750 cm^−1^), ether group (1085 cm^−1^, 1180 cm^−1^) and methyl group (1385 cm^−1^, 2943 cm^−1^, 2995 cm^−1^) were observed. The bands at 1080 and 1185 cm^−1^ and at 1453 and 1382 cm^−1^ corresponded to C-O stretching and C-H bending vibrations, respectively [4,6,7]. Amide peaks (1640 cm^−1^ and 1542 cm^−1^) representing gelatin were observed when gelatin was blended with PLLA. The peak intensities of amides representative of gelatin intensified with increasing gelatin concentration. However, no new peaks generated by the chemical reaction were found, indicating that PLLA and gelatin molecular chains interacted only via van der Waals forces [4,6,7]. In tissue engineering, cells can be grown on scaffolds implanted in organ defects. When implanted in vivo, PLLA/gelatin membranes can simply degrade over time through the hydrolysis of ester bonds [14,15,38,39,40,41]. The degradation of PLLA/gelatin was further enhanced by the localization of extracellular degradative enzymes on the PLLA surface. The cleavage of ester bonds in PLLA, formation of lactic acid oligomers and monomers, and fragmentations on PLLA enhance enzymatic degradation and cause the digestion of PLA oligomers and monomers by bacterial cells [14]. In PLLA/gelatin blends, biodegradation may be more pronounced in PLLA phases with a higher gelatin content due to increased hydrolysis [14,15], as depicted in Figure 5. Abiotic hydrolysis is known to be the rate-limiting step in the degradation process rather than enzymatic degradation [15]. The degradation rate increased dramatically with increased gelatin content. However, the shape of the semi-crystalline PLLA/gelatin blend remained unchanged due to homogeneous degradation [14,15,38,39,40,41]. It was reported that the rate of degradation depends mainly on the crystallinity and molecular weight [14,15]. No appreciable degradation of PLLA (less than 3%) was observed with increasing time, as depicted in Figure 5. The degradation rate of PLLA/gelatin rapidly increased for the 1st week, then decreased until the 4th week, reaching a plateau. When 11 wt% gelatin was added to PLLA, 2% degradation of the PLLA/gelatin blend occurred just after incubation for 1 week at 37 °C in 0.01 M PBS solutions containing 0.1 mg/mL of lysozyme. However, the degradation rate reached a plateau of 9% after 4 weeks. As 11, 25, 30 and 40 wt% of gelatin was added to PLLA, the degree of degradation was extended from 3% to 9%, 19%, 26% and 38%, respectively, as displayed in Figure 5. SEM images (Figure S7) of the membrane surface showed that minor and intermittent aggregation by water and enzymes could be seen in 3 wt% PLLA after 8 weeks in the medium. Aggregates began to gather and clump together as the gelatin content increased to 25%. The membrane broke down in the form of a crater and began to decompose as the gelatin content exceeded 30 wt%. The surface deterioration was noticeable as if it was coated with a thin film in the fibrous layer, which means that the degree of decomposition intensified dramatically as the gelatin content increased to 40 wt%. Cross-sectional images of the membranes exhibited that the morphology changed from dense nanofibrous structure to cleavage and fragmentation of fibers as the gelatin content increased from 0 to 40 wt%. The increased hydrolysis cleaved the ester bonds of PLLA, resulting in severe degradation of the gelatinous membrane [14,15]. The rate of biodegradation of PLLA/gelatin blends could be tuned by adjusting the gelatin content. Therefore, it could be concluded that the addition of gelatin was the most influential parameter for the degradation of PLLA/gelatin blends.

### 3.5. Cell Viability and Proliferation

**Figure 6:**
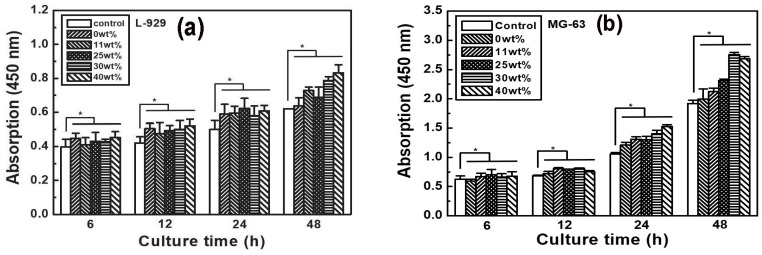
Proliferation of (**a**) L-929 and (**b**) MG-63 cells on the negative control and various PLLA/gelatin membranes with time. All experiments were performed in triplicate. Results were expressed as mean ± standard deviation (* *p* < 0.05).

**Figure 7:**
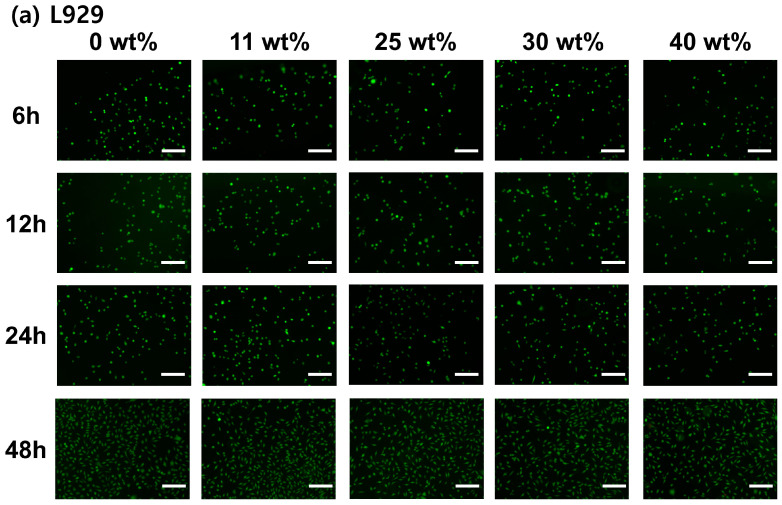

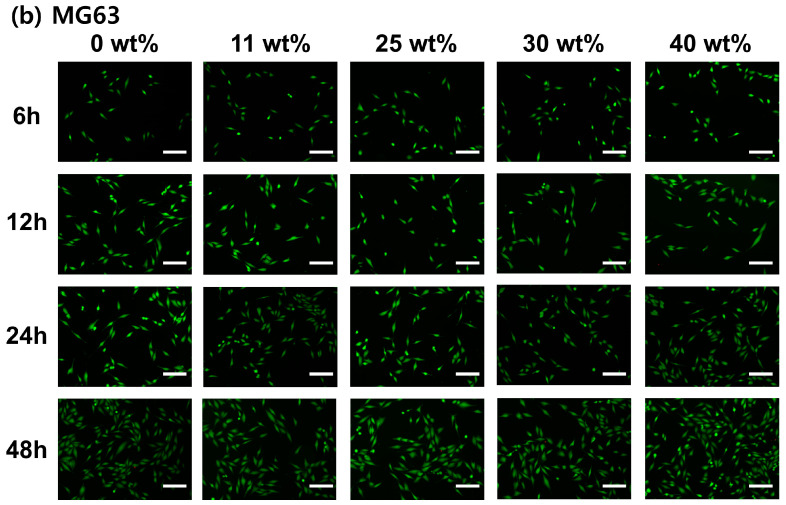
Live/Dead assays showing (**a**) L-929 and (**b**) MG-63 cells incorporated in PLLA/gelatin membranes containing different gelatin content after 6, 12, 24 and 48 h. Note that the scale bar is 200 μm and amplification is 10×.

The cytotoxicity of PLLA/gelatin membranes with gelatin concentrations ranging from 0 to 40% determines the toxicity [7,30]. The L-929 and MG-63 cell viabilities on PLLA/gelatin membranes containing 0%, 11%, 25%, 30% and 40% gelatin were 114%, 111%, 110%, 114% and 108% and 97%, 97%, 97%, 101% and 98%, respectively, compared with the negative control. All membranes were not cytotoxic under the conditions of this study. The results of cell proliferation of L-929 and MG-63 cells in the PLLA/gelatin membranes are displayed in Figure 6, showing that the L-929 and MG-63 cells adhered well to the membrane regardless of the gelatin ratio of the PLLA/gelatin membrane. Cell viability was further examined over the incubation time (6, 12, 24 and 48 h) using the Live/Dead assay kit [33]. The viable L-929 and MG-63 cells (stained green) and dead cells (stained red) were visualized by fluorescence microscope. As can be seen in Figure 7, red cells were not observed at all incubation times. Viable L-929 and MG-63 cells stained green were observed throughout the membrane, showing significant cell proliferation over more than 24 h of culture. It proliferated continuously over time, suggesting that it is suitable for applications in membranes for tissue and bone regeneration [8,30,31]. The PLLA/gelatin membrane containing gelatin (10~40%) is suitable as a barrier membrane for absorbable GTR because of its marketed mechanical properties and biocompatibility [7,31]. The synergic combination of structural integrity and modified hydrophilicity may be effective for periodontal barrier biomaterials requiring fast healing regeneration [26,30,31].

## 4. Discussion

PLA is produced from agricultural resources, such as corn [1,4,6,14]. There are three optical isomers of lactide including L-lactide, D-lactide and DL-lactide. Equimolar racemic mixtures between PLLA and PDLA enantiomers were demonstrated to yield stereo polymer blends of PLLA and PDLA with tensile strength, elastic modulus and elongation at break and biodegradability [14,24]. PLLA and PDLA have similar chemical properties, but PDLLA has different properties because of its crystal structure. Amorphous PDLLA experiences shrinkage during incubation in PBS at 37 °C. PLA can be copolymerized with poly(glycolic acid) (PGA) [10,11,26,42,43,44]. PLGA shows biocompatibility and controllable biodegradability, degradation rate, hydrophilicity and mechanical properties [11]. However, PLGA requires a coating of bioactive substances to improve cell adhesion by providing a biomimetic interface between the polymer and cells [11,41,42,43,44]. The degradation rate and crystal structure of PLGA were controlled by adjusting the composition ratio of LA/GA. PLGA is available in fully amorphous or highly crystalline forms, making it suitable for drug-delivery systems and bone-grafting materials [11]. The hydrophilicity of BP can be controlled by blending with hydrophilic polymers and plasma treatments. Plasma techniques, such as sputtering and plasma etching, are versatile in controlling the wettability and topography of membranes [8,11]. Increased surface roughness can promote the proliferation and migration of osteoblast cells. The grooved surface inhibited the proliferation and migration of epithelial cells. BP membranes with proper surface topographies, hydrophilicity and biodegradability can ensure better GTR. In addition, controlled drug delivery and gene expression are known to be effective in cancer therapy, wound healing, cell migration, cell modification, and reproductive and regenerative medicine. PLA microchambers (MCs) allow for smart encapsulation (Rhodamine B, doxycycline) for precise release via the photothermal opening of isolated MCs [45]. However, this is beyond the scope of this study. The barrier membrane combined with the bone graft around the implant can be further functionalized by loading it with growth factors (platelet-derived growth factor, bone morphogenetic protein, recombinant human growth/differentiation factor-5), antibiotics (tetracycline, metronidazole) and regenerative cells (adipose-derived stromal cell) for complete GTR [11,18,42,43,44,45,46,47,48,49].

A membrane requires full in situ functionality for 4 to 6 weeks for GTR and 16 to 24 weeks for guided bone regeneration (GBR) [11]. It is recommended to maintain integrity for at least 12 weeks. Among BPs, PLLA is widely used in biomedical applications due to its biodegradability, biocompatibility and mechanical properties. Semi-crystalline PLLA can be used in an alloy with metals, inorganic compounds and/or natural polymers [1,2,3,4,5,6,7,8,9,10,11]. Membranes reinforced with metals (zinc, magnesium, iron, strontium), inorganic compounds (hydroxyapatite, calcium phosphate, bioactive glass) or natural polymers (polysaccharides, proteins: collagen, gelatin) may offer an advantageous alternative in GTR [1,2,3,4,5,6,7,8,9,10,11,26,35]. Alloy barriers combined with bone grafts [11,18] containing growth factors [46,47], antibiotics [48] and regenerative cells [38,49] were proposed for future GTR/GBR, but further in vivo testing is needed.

Allograft absorbable periodontal-tissue-regeneration-inducing material is manufactured by decellularizing skin collected from a donated carcass [26]. Dentists are reluctant to use decellularized human skin as a barrier material for periodontal tissue regeneration due to its low strength. The pericardium-derived or peritoneal-derived xenograft tissue regeneration inducer is collected from an animal (cattle or pig). However, they are expensive and the quantity is small [7,26]. Commercial collagen products have low elongation, poor hydration and are expensive. A hybrid polymer was investigated to develop a low-cost absorbable synthetic graft inducer for periodontal regeneration while satisfying market demand. As a biodegradable polymer, a PLLA membrane was evaluated because of its excellent mechanical properties, morphological similarity to the ECM, cell adhesion and proliferation, and biodegradability. PLLA at 3 wt% was chosen because of its experimentally optimized mechanical properties. The strength, failure strain and WUC of 3 wt% PLLA were 9.1 ± 0.9 MPa, 3.0 and 329%, respectively. The hydrophilicity of PLLA has been improved by alloying it with a natural polymer. In this study, gelatin was added to PLLA up to 40 wt%. As the gelatin content increased from 0% to 11%, the strain at break decreased from 3.0 to 1.5, but WUC and WCA increased and decreased dramatically from 329% to 907% and from 127 ± 1.2° to 0°, respectively. The failure strain of the dry PLLA/gelatin membranes decreased dramatically to less than 1.0 when the gelatin doping was above 25%. Periodontal barrier membranes and sutures were used in wet conditions. The strain of the wet PLLA/gelatin membranes increased from 0.5 to 2.1 as gelatin was blended with PLLA at 40 wt%. The wet PLLA/gelatin membranes had a slight decrease in strength but they were sufficient for GTR [7,26], as depicted in Figure S5. The degree of degradation can be adjusted by blending the PLLA and gelatin content. Enhanced degradation can be achieved by using low-molecular-weight PLLA and a higher content of hydrophilic polymer. The use of low-molecular-weight PLLA and a higher content of hydrophilic polymers may enhance the degradation rate. The excellent mechanical properties, WUC, WCA, biodegradability, cytotoxicity, cell proliferation and in vitro cell viability suggested that the PLLA/gelatin blend is highly suitable as a barrier for absorbable GTR. However, additional research is needed to test in vivo alloy barriers combined with bone graft materials containing growth factors, antibiotics and regenerative cells.

## 5. Conclusions

PLLA and PLLA/gelatin membranes were prepared to evaluate the potential of the membranes for absorbable GTR. The optimal PLLA composition of the membrane was 3 wt% and the strength and strain were 9.1 ± 0.9 MPa and 3.0 ± 0.1, respectively. The tensile strength, WUC and WCA of the PLLA/gelatin membrane changed from 9.1 ± 0.9 MPa to 13.3 ± 2.3 MPa, from 329% to 1248% and from 127 ± 1.2° to 0°, respectively, as the gelatin content increased from 0 wt% to 40 wt%. However, the failure strain dramatically decreased from 3.0 to 0.5. Commercial membranes were used wet. Wet stress and strain of PLLA/gelatin membranes in water decreased from 13.3 ± 2.3 MPa to 6.3 ± 0.3 MPa and increased from 0.5 to 2.1, respectively, as the gelatin was blended with PLLA at 40 wt%. The biodegradation rate of the PLLA/gelatin blend increased to 38% as the gelatin content increased to 40%. The L-929 and MG-63 cell viabilities of the PLLA/gelatin membranes were always above 95%. The experimental results suggest that the tunable PLLA/gelatin hybrid membrane was well suited for absorbable GTR. Hence, the studied barrier membranes based on gelatin blended PLLA and combined with bone graft were effective at improving the healing of such important fibrous connective tissue, bone tissue and marrow tissue. However, further studies are needed for in vivo testing of combined bone graft and hydrophilic barrier membranes containing growth factors, antibiotics and regenerative cells for comprehensive clinical evaluation.

## Data Availability

The data presented in this study can be found in the article or Supplementary Materials.

## Supplementary Materials

The following supporting information can be downloaded from https://www.mdpi.com/article/10.3390/polym15183844/s1. Figure S1: Mechanical properties of various PLLA membranes. Figure S2: Mechanical properties of various PLLA/gelatin membranes. Figure S3: Optical photographs of various PLLA/gelatin membranes immersed in water and water droplets after 5 s of contact with various PLLA/gelatin membranes. Figure S4: Finger-stretched optical photographs of various PLLA/gelatin membranes in dry and wet conditions. Figure S5: (a) Strength and (b) strain at break of various PLLA/gelatin membranes in dry and wet conditions. Figure S6: A schematic diagram of biodegradation of PLLA/gelatin membrane. Figure S7: SEM images (600×) of surface and cross-section of PLLA/gelatin membranes degraded in a medium for 8 weeks at different gelatin concentrations: (a) 0%, (b) 11%, (c) 25%, (d) 30% and (e) 40%. Note that the scale bar is 25 μm.
