# Supplementary material for: Electrospun Poly(L-Lactic Acid)/Gelatin Hybrid Polymer as a Barrier to Periodontal Tissue Regeneration
Source: Polymers (Basel). 2023 Sep 21;15(18):3844. doi: 10.3390/polym15183844 (PMC10537136; doi:10.3390/polym15183844)
Supplement: Supplementary file 1 [file polymers-15-03844-s001.zip › polymers-2579913-supplementary.pdf]

Support Information of: Electrospun Poly(L-lactic acid)/Gelatin Hybrid Polymer as A Barrier to Periodontal Tissue Regeneration

Youngchae Cho [1], Heeseok Jeong [1], Baeyeon Kim [2], Juwoong Jang [3], Yo-Seung Song [4,†] and Deuk Yong Lee [1,*,†]

[1] Department of Biomedical Engineering, Daelim University, Anyang 13916, Korea; youngchae6744@naver.com (Y.C.); hsjeong@daelim.ac.kr (H.J.); duke1208@gmail.com (D.Y.L.)
[2] Department of Materials Science and Engineering, Incheon National University, Incheon 22012, Korea; bykim@incheon.ac.kr
[3] Department of R&D Center, Renewmedical Co., Ltd., Bucheon 14532, Korea; orienta@empas.com
[4] Department of Materials Science and Engineering, Korea Aviation University, Goyang 10540, Korea; yssong@kau.ac.kr
* Correspondence: duke1208@gmail.com (D.Y.L.); yssong@kau.ac.kr (Y.S.)

Supporting Figures:

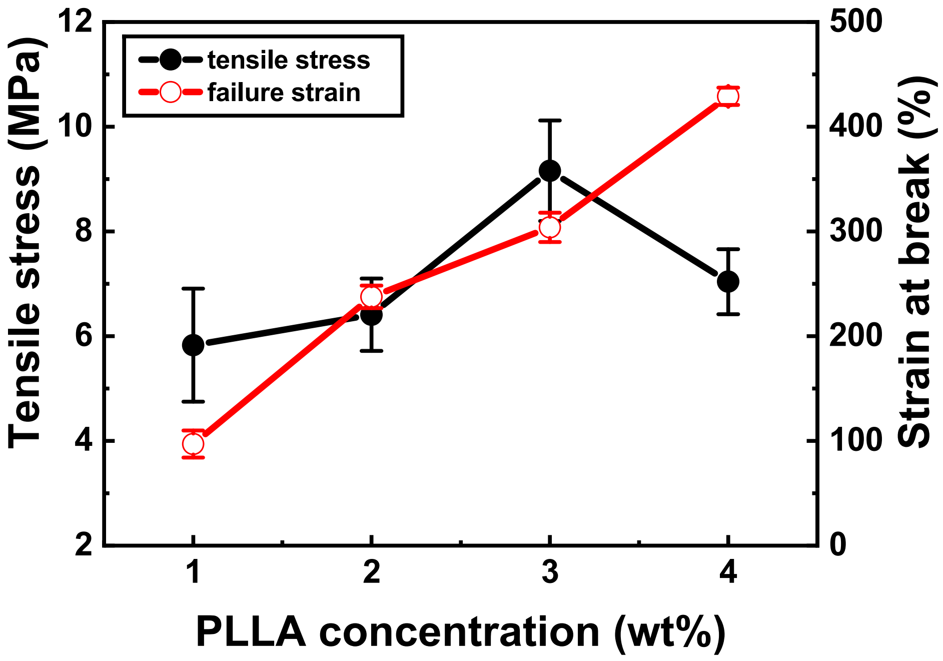

Figure S1. Mechanical properties of various PLLA membranes.

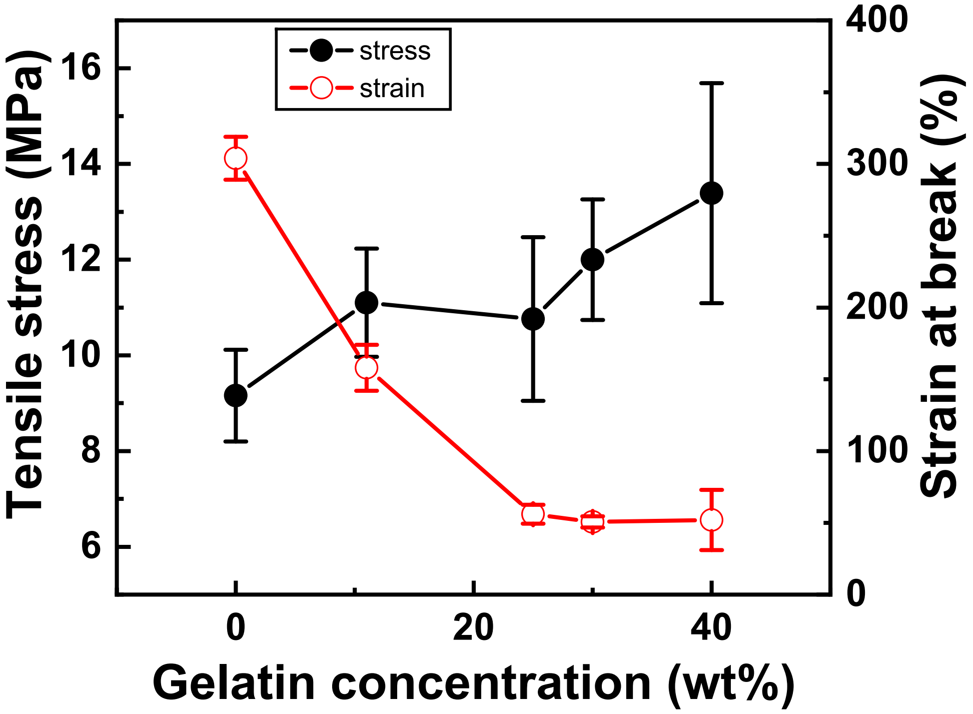

Figure S2. Mechanical properties of various PLLA/gelatin membranes.

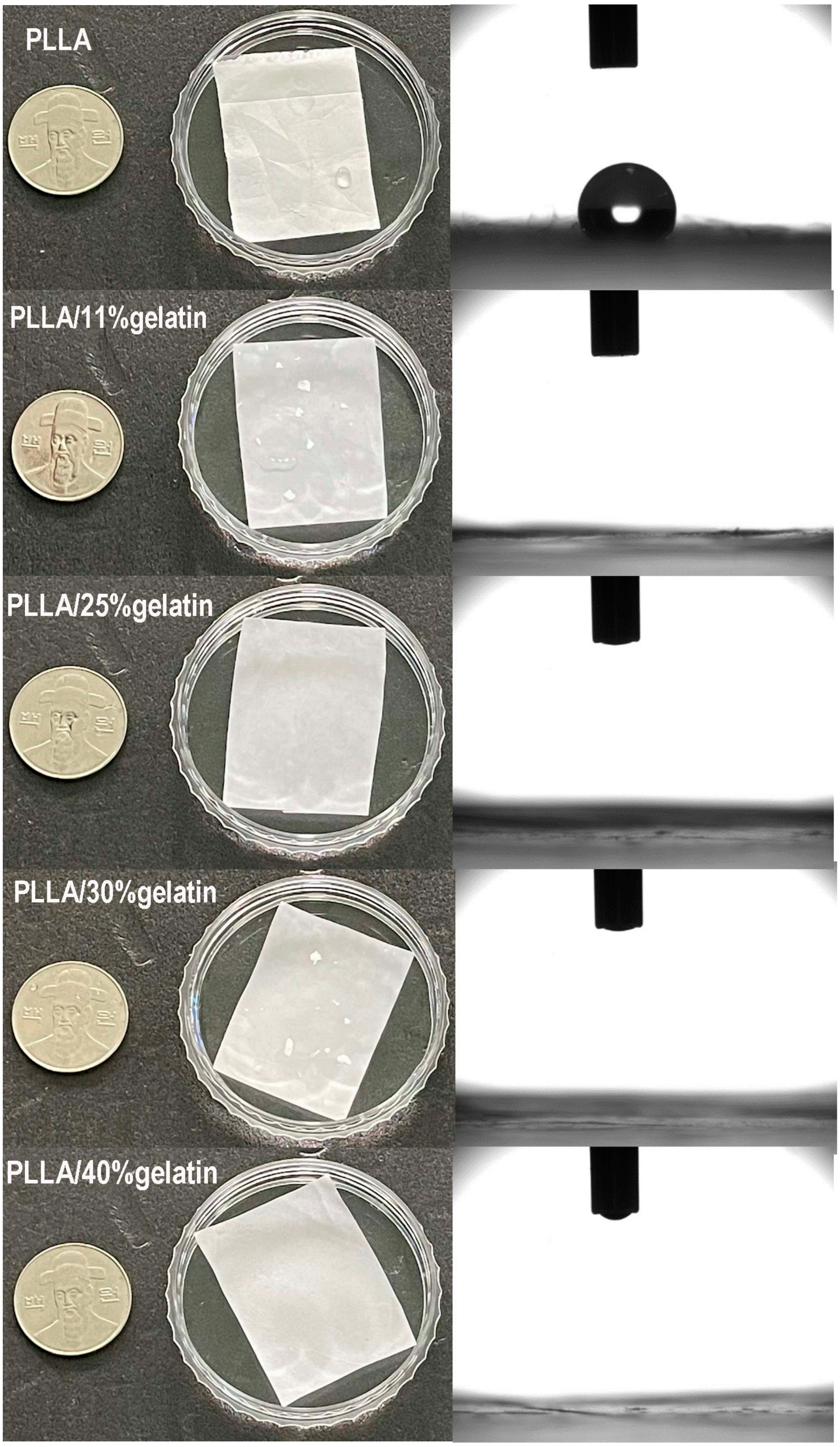

Figure S3. Optical photographs of various PLLA/gelatin membranes soaked in water and water droplets after 5 s of contact with various PLLA/gelatin membranes.

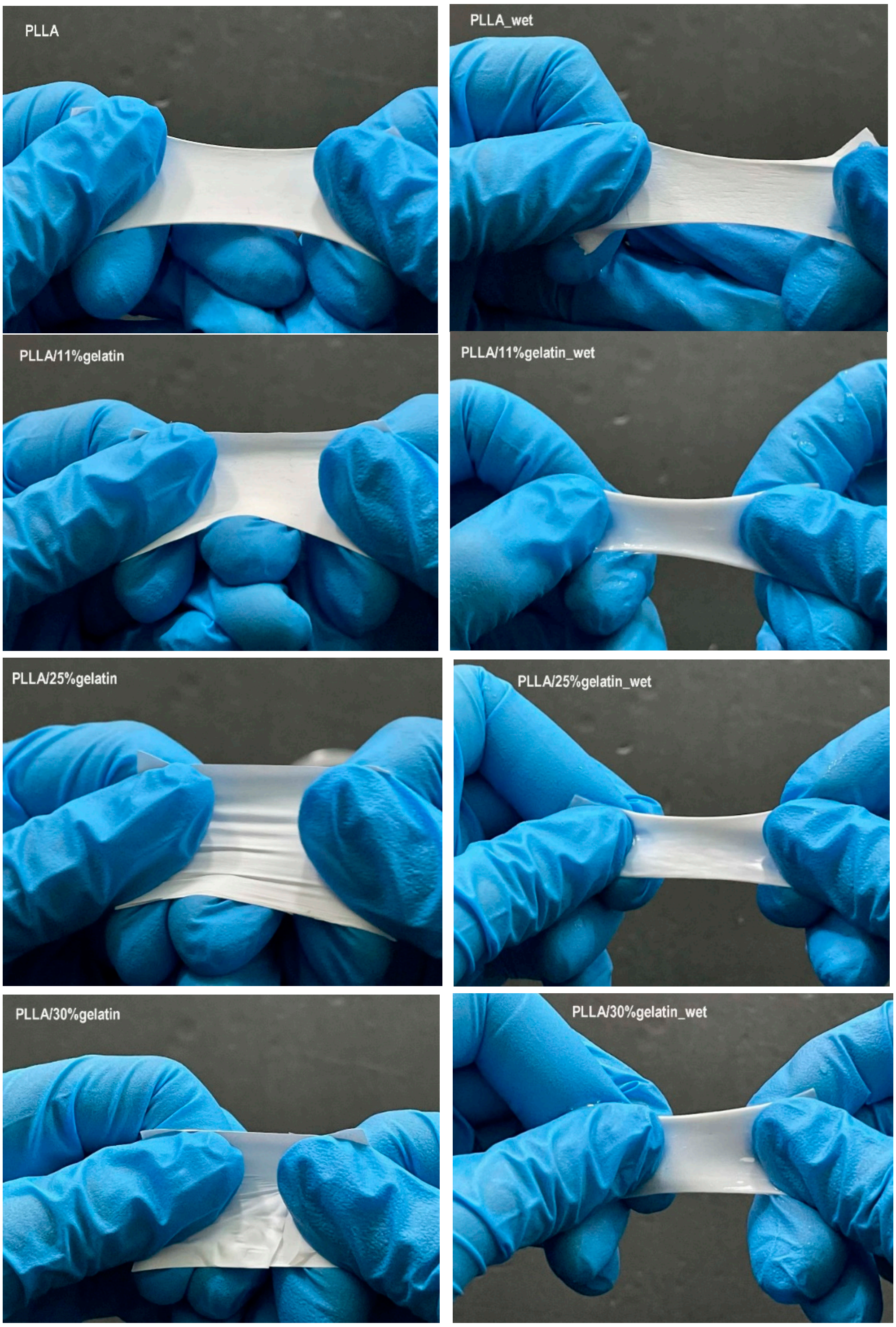
PLLA
PLLA_wet
PLLA/11%gelatin
PLLA/11%gelatin_wet
PLLA/25%gelatin
PLLA/25%gelatin_wet
PLLA/30%gelatin
PLLA/30%gelatin_wet

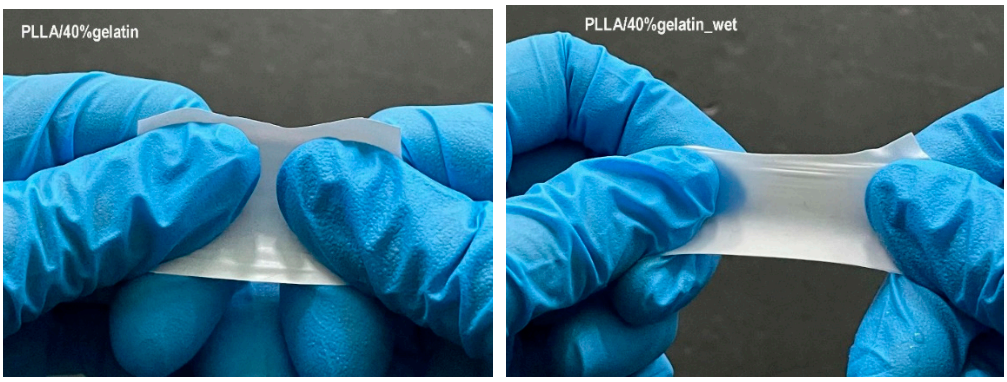

Figure S4. Finger stretched optical photographs of various PLLA/gelatin membranes in dry and wet conditions.

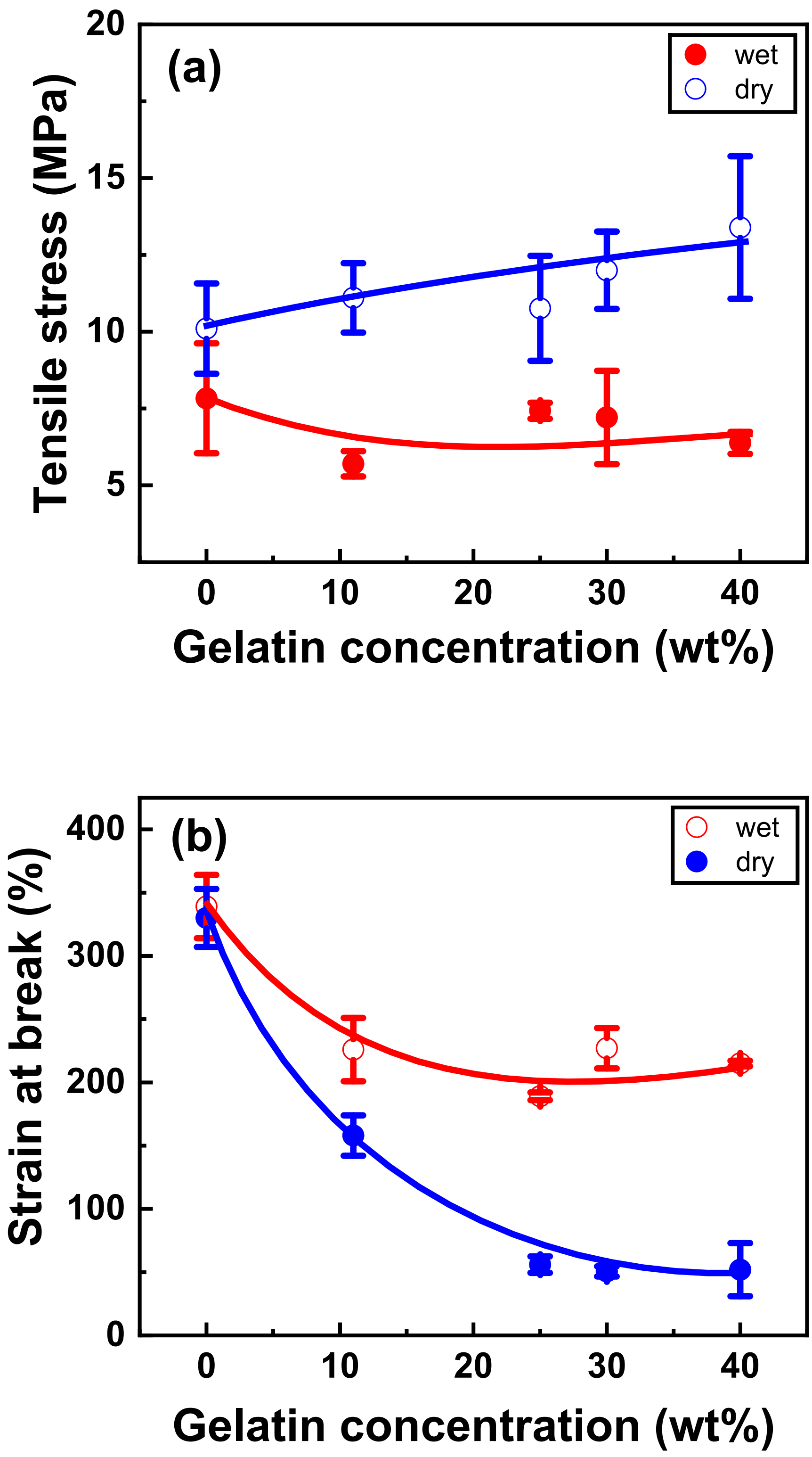

Figure S5. (a) Strength and (b) strain at break of various PLLA/gelatin membranes in dry and wet conditions.

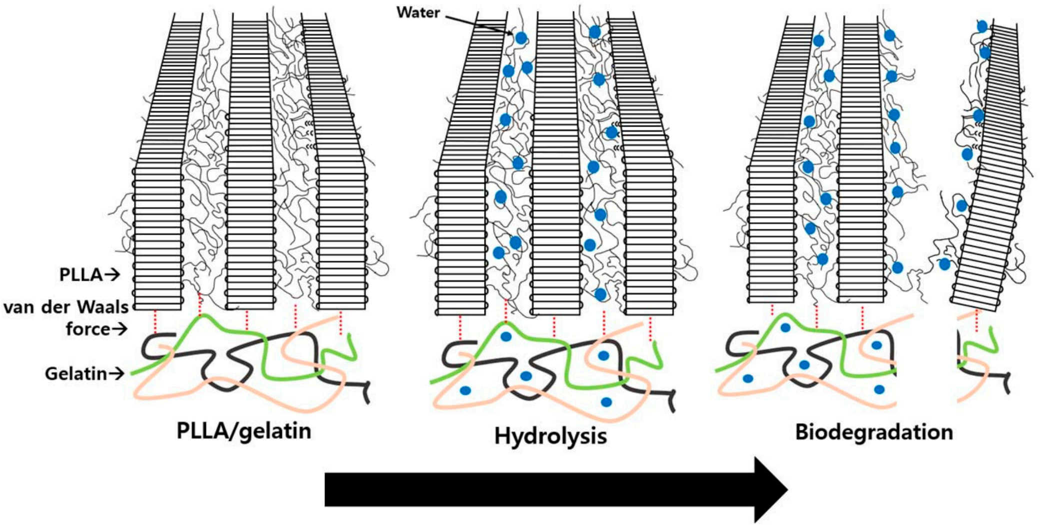

Figure S6. A schematic diagram of biodegradation of PLLA/gelatin membrane.

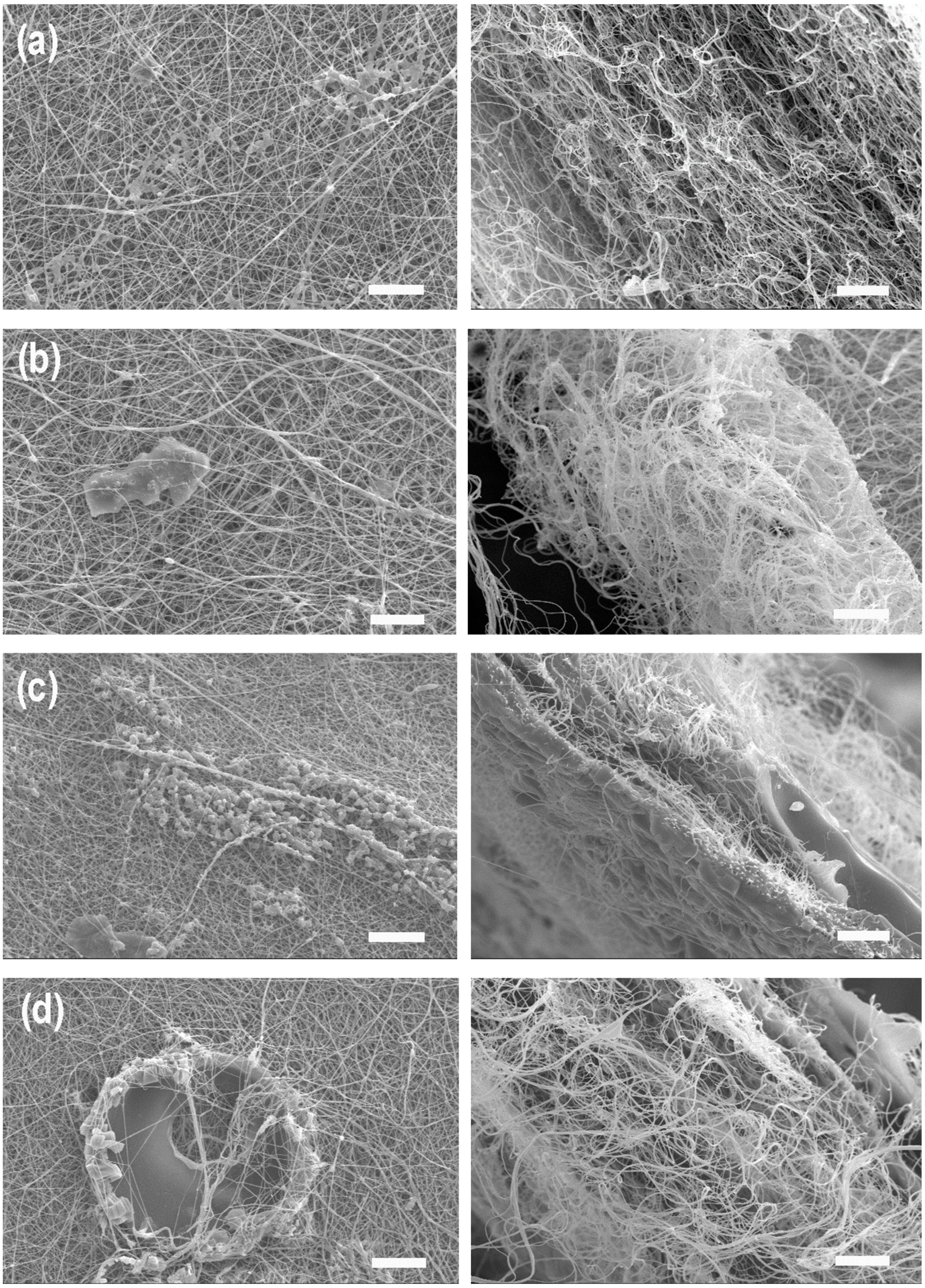
(a)
(b)
(c)
(d)

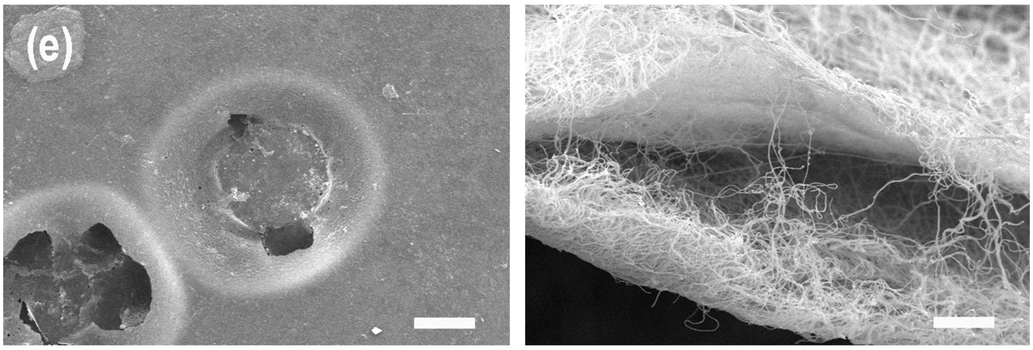

Figure S7. SEM images (×600) of surface and cross-section of PLLA/gelatin membranes degraded in medium for 8 weeks at different gelatin concentrations: (a) 0%, (b) 11%, (c) 25%, (d) 30% and (e) 40%. Note that the scale bar is 25 μm.
